# Effects of Silver Nanoparticles on Physiological and Proteomic Responses of Tobacco (*Nicotiana tabacum*) Seedlings Are Coating-Dependent

**DOI:** 10.3390/ijms232415923

**Published:** 2022-12-14

**Authors:** Renata Biba, Petra Cvjetko, Mirta Tkalec, Karla Košpić, Petra Peharec Štefanić, Sandra Šikić, Ana-Marija Domijan, Biljana Balen

**Affiliations:** 1 Department of Biology, Faculty of Science, University of Zagreb, Horvatovac 102a, 10000 Zagreb, Croatia; 2 Department of Ecology, Institute of Public Health “Dr. Andrija Štampar”, Mirogojska cesta 16, 10000 Zagreb, Croatia; 3 Department of Pharmaceutical Botany, Faculty of Pharmacy and Biochemistry, University of Zagreb, Ante Kovačića 1, 10000 Zagreb, Croatia

**Keywords:** silver nanoparticles, coatings, reactive oxygen species, oxidative stress, antioxidative enzymes, nonenzymatic antioxidants, protein expression

## Abstract

The harmful effects of silver nanoparticles (AgNPs) have been confirmed in many organisms, but the mechanism of their toxicity is not yet fully understood. In biological systems, AgNPs tend to aggregate and dissolve, so they are often stabilized by coatings that influence their physico-chemical properties. In this study, the effects of AgNPs with different coatings [polyvinylpyrrolidone (PVP) and cetyltrimethylammonium bromide (CTAB)] on oxidative stress appearance and proteome changes in tobacco (*Nicotiana tabacum*) seedlings have been examined. To discriminate between the nanoparticulate Ag form from the ionic one, the treatments with AgNO_3_, a source of Ag^+^ ions, were also included. Ag uptake and accumulation were found to be similarly effective upon exposure to all treatment types, although positively charged AgNP-CTAB showed less stability and a generally stronger impact on the investigated parameters in comparison with more stable and negatively charged AgNP-PVP and ionic silver (AgNO_3_). Both AgNP treatments induced reactive oxygen species (ROS) formation and increased the expression of proteins involved in antioxidant defense, confirming oxidative stress as an important mechanism of AgNP phytotoxicity. However, the mechanism of seedling responses differed depending on the type of AgNP used. The highest AgNP-CTAB concentration and CTAB coating resulted in increased H_2_O_2_ content and significant damage to lipids, proteins and DNA molecules, as well as a strong activation of antioxidant enzymes, especially CAT and APX. On the other hand, AgNP-PVP and AgNO_3_ treatments induced the nonenzymatic antioxidants by significantly increasing the proline and GSH content. Exposure to AgNP-CTAB also resulted in more noticeable changes in the expression of proteins belonging to the defense and stress response, carbohydrate and energy metabolism and storage protein categories in comparison to AgNP-PVP and AgNO_3_. Cysteine addition significantly reduced the effects of AgNP-PVP and AgNO_3_ for the majority of investigated parameters, indicating that AgNP-PVP toxicity mostly derives from released Ag^+^ ions. AgNP-CTAB effects, however, were not alleviated by cysteine addition, suggesting that their toxicity derives from the intrinsic properties of the nanoparticles and the coating itself.

## 1. Introduction

Engineered nanomaterials (ENMs) with dimensions on the nanoscale (1–100 nm) are designed and manufactured to enhance the properties of their bulk materials, such as strength, flexibility, conductivity, ultraviolet (UV) protection and antimicrobial effects [1,2]. Silver nanoparticles (AgNPs), which make up around one fifth of all ENM-containing products on the market today [3], can be found in various textiles, household products, cosmetics and food packaging [4], with a growing increase in demand as new applications are being discovered. The enhanced antibacterial [5,6,7], antifungal [8,9], antiviral [10,11] and sporicidal [12] activity of AgNPs makes them particularly interesting for medical, environmental and agricultural use. However, increased consumption and release of AgNPs into the environment raise concerns about their safety and risks for the ecosystems and human health. With studies already showing higher concentrations of AgNPs in soil compared to water or air [13], plants are likely to accumulate AgNPs and act as their primary source in the food chains [14].

The fate of released AgNPs is determined by the properties of the environment (i.e., electrolyte composition, ionic strength, pH and natural organic matter content) as well as the physico-chemical properties of the AgNPs themselves [3,15]. The shape, size and surface coating of AgNPs greatly influence their physical interactions with plant cells and ultimately determine their toxicity [4]. Although numerous research papers suggest AgNP size is a critical determinant of their phytotoxicity (reviewed in [4]), newer research also emphasizes the importance of the surface coating used for AgNP stabilization. The functionalization of AgNP surfaces with coatings that exploit steric or electrostatic repulsion can significantly reduce the aggregation and dissolution of silver ions (Ag^+^) from their surface and in a way that stabilizes AgNPs in a suspension [15,16]. However, the addition of such coatings also modifies their biological activity and can lead to differential plant response [17,18,19]. Namely, the chemical composition and charge of the used coating determine AgNP interactions with biomolecules. It was found that electrostatically stabilized AgNPs, with either positive or negative overall charge, exhibit generally more negative effects on plant growth and physiology compared to sterically stabilized and uncharged AgNPs [20,21,22,23,24]. On top of that, several authors suggested that the coating alone can induce phytotoxic effects. Treatments with cationic surfactants cetyltrimethylammonium bromide (CTAB) and dimethyldidodecylammonium bromide (DDAB) led to significant inhibition of germination and early growth [21,22], while the same was not observed in treatments with polymeric molecules, such as polyvinylpyrrolidone (PVP) and polyethylene glycol (PEG) [22,25,26], indicating that the coating itself can contribute to the overall AgNP toxicity.

Significant changes observed in plants after different AgNP treatments, both at a morphological and physiological level, indicate the severe cytotoxic effect of AgNPs that can result in the damage of cell metabolism, oxidative stress and, eventually, cell death [27]. Numerous studies have shown that the primary mechanism underlying AgNP toxicity in plants arises from the excessive production of reactive oxygen species (ROS) and the consequent oxidative damage of important biomolecules, resulting in protein modification, DNA impairment and lipid peroxidation [28,29]. Namely, internalized AgNPs can act as a reducing agent, increasing plants’ cellular ROS content directly by electron donation to molecular oxygen [30] or through Fenton- or Haber-Weiss-like reactions [31,32,33]. Moreover, AgNPs can further promote ROS accumulation by damaging chloroplast and mitochondrial function, leading to the disturbance of electron transport and increasing the NADP^+^/NADPH ratio [33,34]. On top of that, Ag^+^ ions released from the AgNP surface can deactivate cellular enzymes, damage cell membranes and reduce membrane potential, and in that way contribute to the appearance of oxidative stress [30,35]. AgNPs can also interfere with the expression of genes and proteins involved in the stress response of plants; however, genomic and proteomic research is still scarce and limited to either one plant species or one type of AgNPs (reviewed in [17,36]), making it difficult to completely elucidate the mechanism of defense against AgNP phytotoxicity. 

In our previous research, we investigated the effects of AgNP-PVP and AgNP-CTAB on tobacco (*Nicotiana tabacum*) seed germination and early growth [22]. The results showed that both AgNP-PVP and AgNP-CTAB treatments induced dose-dependent increase in measured silver content, indicating that the coating used for AgNP stabilization had no effect on uptake and accumulation of AgNPs in the plant tissue. However, AgNP-CTAB caused severe negative effects on germination and growth parameters, while the effects of AgNP-PVP were far less severe and only significant at the highest tested concentration, suggesting that the coating plays an important role in the mechanism of AgNP phytotoxicity. Treatments with the CTAB coating itself also exerted negative effects on tobacco seedlings, substantiating the importance of coating in AgNP toxicity. Moreover, the addition of cysteine, a strong ligand of Ag^+^ ions [37], alleviated AgNP-PVP-induced effects, but failed to improve germination and growth parameters after AgNP-CTAB treatment, suggesting that the toxicity of AgNP-PVP arises from the release of Ag^+^ ions, while AgNP-CTAB toxicity can be ascribed to surface coating itself. 

Considering the observed differences in growth and germination of tobacco seedlings after treatment with differently coated AgNPs, the aim of this study was to reveal possible diversity in coating-dependent mechanisms of toxicity. To achieve that we investigated and compared the effects of AgNPs stabilized with two different coatings, PVP and CTAB, on oxidative stress appearance and proteome changes in tobacco seedlings. To distinguish if the toxicity is caused by nanoparticle form or the ionic form of Ag, we introduced treatment with AgNO_3_, a source of Ag^+^ ions, as well as combined treatments of AgNPs or AgNO_3_ with cysteine, a strong ligand of Ag^+^ ions. Moreover, to ascertain the impact of the coating molecules themselves, treatments with PVP and CTAB alone were also carried out. In order to achieve this aim, we firstly determined the characteristics and stability of differently coated AgNPs in the exposure medium. To gain a deeper insight into the physiological responses and molecular patterns of tobacco seedlings exposed to differently coated AgNPs, we executed an extensive survey on ROS formation and oxidative stress parameters, as well as on the mobilization of enzymatic and non-enzymatic components of the antioxidant machinery. Ultimately, molecular responses were revealed by identifying changes in seedlings proteome. This type of a thorough physiological and molecular study, which included two differently coated AgNPs and AgNO_3_, applied either alone or in combinations with cysteine, as well as the coating molecules themselves, represents a novel approach to the study of AgNP-induced phytotoxic effects. The obtained results can be applied to estimate the possibility of use and safety of coatings applied for stabilization of AgNPs implemented in the agricultural products, such as fertilizers and pesticides, used in cultivation of economically interesting plants.

## 2. Results

### 2.1. Synthesis, Characterization and Stability of AgNP-PVP and AgNP-CTAB in Nutrient Medium

Spectroscopic characterization of AgNP-PVP and AgNP-CTAB upon synthesis showed surface plasmon resonance (SPR) peaks at 412 nm and 443 nm, respectively, confirming the existence of AgNPs in the suspensions with approximate sizes of 40 (AgNP-PVP) and 70 nm (AgNP-CTAB), as previously reported [22]. The bimodal distribution of hydrodynamic diameters (d_H_) was revealed in the AgNP-PVP suspension, with 41.53 ± 1.14 nm and 8.16 ± 0.37 nm, while trimodal distribution characterized AgNP-CTAB suspension, with 64.75 ± 7.28 nm, 26.28 ± 6.37 nm and 227.99 ± 16.02 nm, as previously reported in [22]. ζ potential for AgNP-PVP stock solution was slightly negative (−1.0 ± 0.6 mV), while AgNP-CTAB had an overall positive charge (11.4 ± 1.1 mV).

The addition of AgNP-PVP into ½ MS medium showed an immediate red-shifting of the SPR peak compared to the stock solution (from 420 to 428 nm), indicating the formation of small agglomerates (Figure S1A). After the initial changes, AgNP-PVP remained stable in the medium for the first 24 h of the measurement. During the next seven days, however, further shift of the SPR peak towards the higher wavelengths was observed, followed by a gradual decrease in absorbance, showing significant agglomeration of AgNP-PVP in the medium (Figure S1B). AgNP-CTAB showed a significant shift of the SPR peak towards the higher wavelengths (from 420 nm to 450 nm) already during the first 60 min of measurement, followed by a fast decrease in the absorbance, showing fast agglomeration of the NPs in the medium (Figure S2A). Measurements following in the next hours showed further agglomeration, and after 24 h, AgNPs could no longer be detected in the medium (Figure S2B). The faster agglomeration of AgNP-CTAB (Figure S4) compared to AgNP-PVP (Figure S3) in the ½ MS medium was further corroborated by DLS measurements. On top of that, the alteration of the AgNP-CTAB surface charge, measured as the immediate change of ζ potential (+11.4 to −7.0 mV), indicated a fast loss of the surface coating (Table S1). 

The addition of cysteine had a disparate effect on the stability of AgNP-PVP (Figure S1C,D) and AgNP-CTAB (Figure S2C,D) in the ½ MS medium. Even though both solutions showed the initial formation of a second peak at the lower wavelengths, indicating the release of Ag^+^ ions from the surface and formation of secondary AgNPs, cysteine ultimately caused the complete dissolution of AgNP-PVP and agglomeration of AgNP-CTAB in the nutrient medium.

### 2.2. Silver Uptake and Localization

Silver uptake exhibited a dose-dependent response upon exposure of tobacco seedlings to either AgNP-PVP and AgNP-CTAB or AgNO_3_ treatments (Table 1). No significant difference in accumulation of silver was recorded between the corresponding concentrations of different treatments. Addition of cysteine resulted in significant decrease in the silver uptake in all combined treatments, which was more pronounced after exposure to combined treatments with AgNO_3_. 

**Figure 1:**
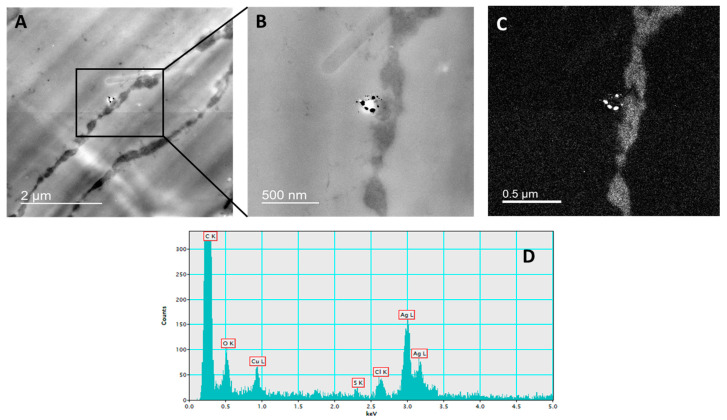
Localization of AgNPs in the root cells of tobacco seedlings treated with 100 μM AgNP-PVP. TEM microphotographs of root cell with AgNPs in the vacuole of the epidermal cell (**A**), enlarged part of epidermal cell with AgNPs (**B**), silver elemental map and (**C**) energy-dispersive X-ray spectrum (**D**).

**Figure 2:**
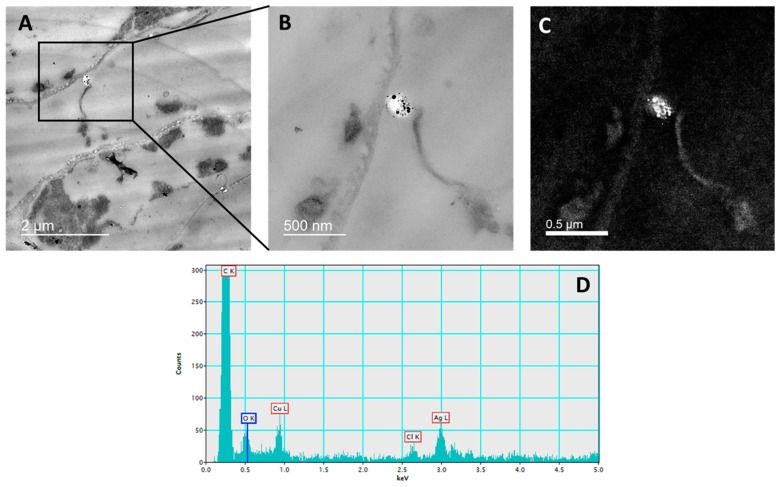
Localization of AgNPs in the root cells of tobacco seedlings treated with 100 μM AgNP-CTAB. TEM microphotographs of root cell with AgNPs in the vacuole near the cell wall of the epidermal cell (**A**), enlarged part of epidermal cell with AgNPs (**B**), silver elemental map and (**C**) energy-dispersive X-ray spectrum (**D**).

The presence of AgNPs in the root cells of tobacco seedlings was confirmed by TEM-EDX analysis after exposure to 100 μM AgNP-PVP (Figure 1) and AgNP-CTAB (Figure 2).

### 2.3. Induction of ROS Formation

**Figure 3:**
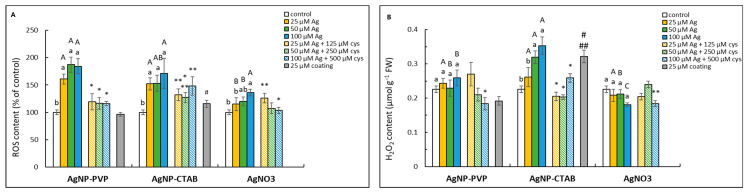
ROS (**A**) and H_2_O_2_ (**B**) contents measured in tobacco seedlings 7 days after exposure to 25, 50 and 100 μM concentration of silver nanoparticles coated with either polyvinylpyrrolidone (AgNP-PVP) or cetyltrimethylammonium bromide (AgNP-CTAB) and of silver nitrate (AgNO_3_) and their combinations with 125, 250 and 500 μM cysteine (cys), as well as 25 μM PVP and CTAB coatings alone. Values represent the means ± standard error of 2 different experiments, each with 6 replicas (n = 12). Treatments significantly different at *p* ≤ 0.05 (one-way ANOVA followed by Duncan post-hoc test) are labelled with different letters; small letters denote the differences among different concentrations of the same treatment type as well as control; capital letters indicate the differences among different types of the treatments of the same concentration; asterisks (*) represent significant differences among treatments with and without cysteine of the corresponding concentration; double asterisks (**) mark significant differences between treatments with cysteine and control; hash signs (#) indicate significant difference between the coatings (PVP and CTAB) and control; and double hash signs (##) represent significant difference between PVP and CTAB coatings.

Both AgNP-PVP and AgNP-CTAB significantly increased ROS content from the lowest applied concentration, while in AgNO_3_ treatment, the increase was only significant at the highest concentration (Figure 3A). The addition of cysteine alleviated AgNP-PVP effects in all combined treatments, but alleviated AgNO_3_ effects only at the highest concentration. H_2_O_2_ content showed a significant increase in 50 and 100 μM AgNP-CTAB treatments, which was partially reduced with the addition of cysteine (Figure 3B). Treatment with CTAB alone also induced both total ROS and H_2_O_2_ formation. 

### 2.4. Oxidative Damage of Biomolecules

**Figure 4:**
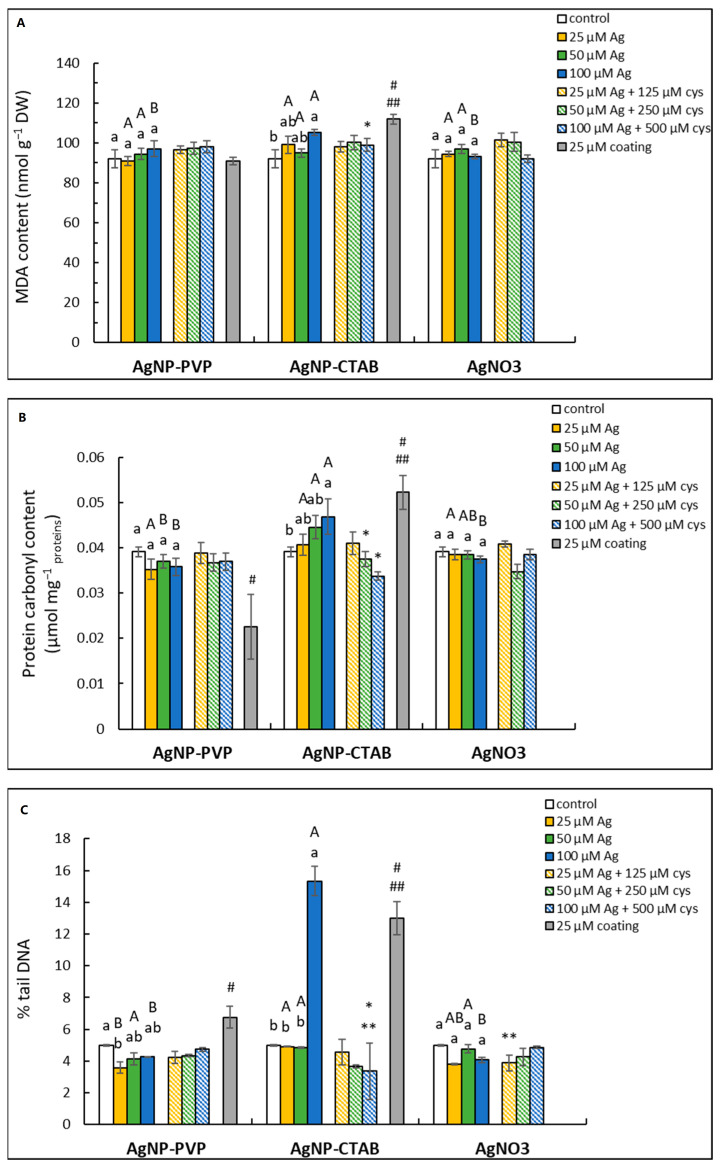
MDA (**A**) and protein carbonyls (**B**) content, as well as % tail DNA (**C**) measured in tobacco seedlings 7 days after exposure to 25, 50 and 100 μM concentrations of silver nanoparticles coated with either polyvinylpyrrolidone (AgNP-PVP) or cetyltrimethylammonium bromide (AgNP-CTAB) and of silver nitrate (AgNO_3_) and their combinations with 125, 250 and 500 μM cysteine (cys), as well as 25 μM PVP and CTAB coatings alone. Values represent the means ± standard error of 2 different experiments, each with 6 replicas (n = 12). Treatments significantly different at *p* ≤ 0.05 (one-way ANOVA followed by Duncan post-hoc test) are labelled with different letters; small letters denote the differences among different concentrations of the same treatment type as well as control; capital letters indicate the differences among different type of the treatment of the same concentration; asterisks (*) represent significant differences among treatments with and without cysteine of the corresponding concentration; double asterisks (**) mark significant differences between treatments with cysteine and control; hash signs (#) indicate significant difference between the coatings (PVP and CTAB) and control; and double hash signs (##) represent significant difference between PVP and CTAB coatings.

Oxidative damage to biomolecules was recorded only upon treatment with 100 μM AgNP-CTAB, which induced an increase in lipid peroxidation (Figure 4A), protein carbonyls (Figure 4B) and % tDNA (Figure 4C) compared to the control, which was alleviated in combined treatments with cysteine.

The CTAB coating alone also induced significant oxidative damage of lipids, proteins and the DNA molecule, while the PVP coating caused only DNA damage, but to a much lesser extent than the CTAB coating. 

### 2.5. Activity of Antioxidative Enzymes

**Figure 5:**
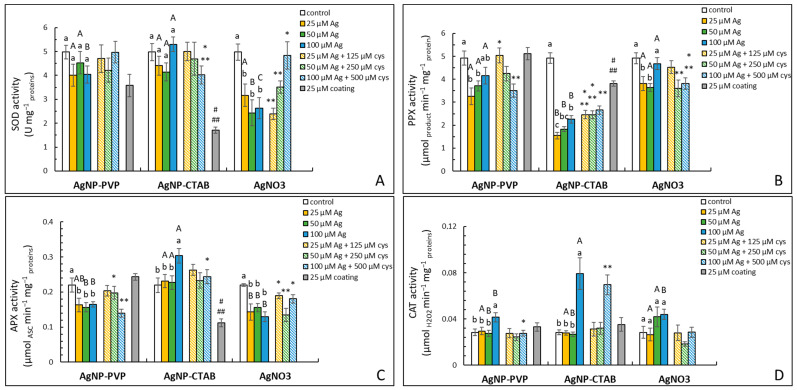
SOD (**A**), PPX (**B**), APX (**C**) and CAT (**D**) activities measured in tobacco seedlings 7 days after exposure to 25, 50 and 100 μM concentration of silver nanoparticles coated with either polyvinylpyrrolidone (AgNP-PVP) or cetyltrimethylammonium bromide (AgNP-CTAB) and silver nitrate (AgNO_3_) and their combinations with 125, 250 and 500 μM cysteine (cys), as well as 25 μM PVP and CTAB coatings alone. Values represent the means ± standard error of two different experiments, each with 6 replicas (n = 12). Treatments significantly different at *p* ≤ 0.05 (one-way ANOVA followed by Duncan post-hoc test) are labelled with different letters; small letters denote the differences among different concentrations of the same treatment type as well as control; capital letters indicate the differences among different type of the treatment of the same concentration; asterisks (*) represent significant differences among treatments with and without cysteine of the corresponding concentration; double asterisks (**) mark significant differences between treatments with cysteine and control; hash signs (#) indicate significant difference between the coatings (PVP and CTAB) and control; and double hash signs (##) represent significant difference between PVP and CTAB coatings.

A significant difference in superoxide dismutase (SOD) activity between ionic and NP silver form was observed. Only AgNO_3_ treatments caused inhibition of SOD activity compared to control and respective AgNP treatments (Figure 5A). Cysteine addition reduced AgNO_3_ effects at the highest concentration of the combined treatment. Interestingly, the CTAB coating alone strongly inhibited SOD activity compared to the control and corresponding PVP treatment. 

As for the activity of peroxidases, AgNP-PVP and AgNO_3_ treatments exhibited similar effects on the pyrogallol peroxidase (PPX) activity with significant inhibition measured after 25 and 50 μM treatments (Figure 5B). PPX activity was only partially recovered in combined treatments of AgNP-PVP or AgNO_3_ with cysteine, but with values lower compared to the control cysteine addition to 100 μM AgNP-PVP, and AgNO_3_ treatments led to a significant decrease in PPX activity compared to its respective treatments without cysteine. AgNP-CTAB exposure also resulted in significant inhibition of PPX activity, which was more prominent compared to the corresponding AgNP-PVP and AgNO_3_ treatments, and only slightly diminished with cysteine addition at 25 and 50 μM concentrations. The CTAB coating also decreased PPX activity. All AgNP-PVP and AgNO_3_ treatments inhibited ascorbate peroxidase (APX) activity compared to the control (Figure 5C), while 100 μM AgNP-CTAB treatment led to a significantly increased value. The addition of cysteine mostly alleviated AgNP-PVP and AgNO_3_-induced effects at lower concentrations, but failed to increase APX activity in combination of 100 μM AgNP-PVP and 500 μM cysteine. In combined 100 μM AgNP-CTAB and 500 μM cysteine treatment, however, observed value was lower compared to AgNP-CTAB treatment alone, and similar to the control. The CTAB coating significantly decreased APX activity compared to control and PVP coating itself. 

Increased CAT activity was measured in all treatment types at the highest applied concentration; significantly higher activity was measured in AgNP-CTAB treatment compared to respective AgNP-PVP and AgNO_3_ treatments (Figure 5D). Combined treatments with cysteine reduced AgNP-PVP- and AgNO_3_-induced effects, even though the decrease measured in the combined AgNO_3_ and cysteine treatment was not statistically significant. Cysteine, however, failed to reduce AgNP-CTAB effects. 

### 2.6. Nonenzymatic Antioxidants

**Figure 6:**
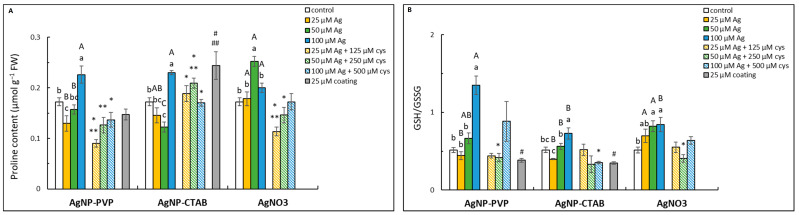
Proline content (**A**) and ratio of reduced and oxidised glutathione (GSH/GSSG) (**B**) measured in tobacco seedlings 7 days after exposure to 25, 50 and 100 μM concentrations of silver nanoparticles coated with either polyvinylpyrrolidone (AgNP-PVP) or cetyltrimethylammonium bromide (AgNP-CTAB) and of silver nitrate (AgNO_3_) and their combinations with 125, 250 and 500 μM cysteine (cys), as well as 25 μM PVP and CTAB coatings alone. Values represent the means ± standard error of 2 different experiments, each with 6 replicas (n = 12). Treatments significantly different at *p* ≤ 0.05 (one-way ANOVA followed by Duncan post-hoc test) are labelled with different letters; small letters denote the differences among different concentrations of the same treatment type as well as control; capital letters indicate the differences among different type of the treatment of the same concentration; asterisks (*) represent significant differences among treatments with and without cysteine of the corresponding concentration; double asterisks (**) mark significant differences between treatments with cysteine and control; hash signs (#) indicate significant difference between the coatings (PVP and CTAB) and control; and double hash signs (##) represent significant difference between PVP and CTAB coatings.

Lower AgNP-PVP and AgNP-CTAB concentrations resulted in the decrease in proline content compared to the control, while 100 μM treatment significantly increased the values (Figure 6A). In AgNO_3_ treatments, only 50 μM concentration significantly increased proline content compared to control and AgNP treatments of the same concentration. In combined treatments with AgNP-PVP, cysteine alleviated the increase in proline content at the highest concentration to a value similar to the control’s, whereas the obtained values after lower concentrations were reduced compared to those obtained after respective treatments without cysteine. Similar observations were recorded in combined treatments of cysteine with 25 and 50 μM AgNO_3_. The addition of cysteine to AgNP-CTAB treatments resulted in significantly increased values in 25 and 50 μM concentrations compared to their respective treatments without cysteine, as well as the control. However, at 100 μM concentration, cysteine addition diminished the AgNP-induced increase in proline content. Exposure to CTAB coating alone, unlike PVP, also resulted in increased value.

The highest concentration of both AgNP treatments induced an increase in GSH/GSSG ratio compared to the control, although it was significantly higher in AgNP-PVP treatment compared to AgNP-CTAB (Figure 6B). GSH/GSSG ratio was also significantly higher after exposure to 50 and 100 μM AgNO_3_ compared to the control, but lower compared to 100 μM AgNP-PVP. The decrease in the GSH/GSSG ratio upon cysteine addition was statistically significant in 100 μM AgNP-CTAB and 50 μM AgNO_3_ treatments. Both PVP and CTAB coatings reduced the GSH/GSSG ratio.

### 2.7. Changes in Proteome

The differential expression of proteins in tobacco seedlings treated with 100 μM concentration of AgNP-PVP, AgNP-CTAB and AgNO_3_ and their combination with 500 μM cysteine are shown in Table 2 and Figure S5. Out of 26 protein spots showing significant difference in protein abundance upon treatments, 21 different proteins were identified in total. AgNP-PVP treatment resulted in 13 proteins with difference in abundance, 10 of which were up-regulated and three down-regulated compared to the control. In combined AgNP-PVP treatment with cysteine, five of the identified proteins were up-regulated, and one was down-regulated compared to the control. AgNP-CTAB treatment changed the abundance of 21 different proteins in total; 17 proteins showed up-regulation, while four were down-regulated compared to the control. Cysteine addition slightly reduced AgNP-CTAB effects, and combined treatment resulted in 11 up-regulated and two down-regulated proteins. AgNO_3_ induced changes in the abundance of 15 proteins in total, 13 of which were up-regulated and two down-regulated compared to the control. Combined treatment of AgNO_3_ and cysteine reduced the number of differently expressed proteins compared to control and treatment with AgNO_3_ alone; five proteins were up-regulated, and one was down-regulated compared to control. 

**Figure 7:**
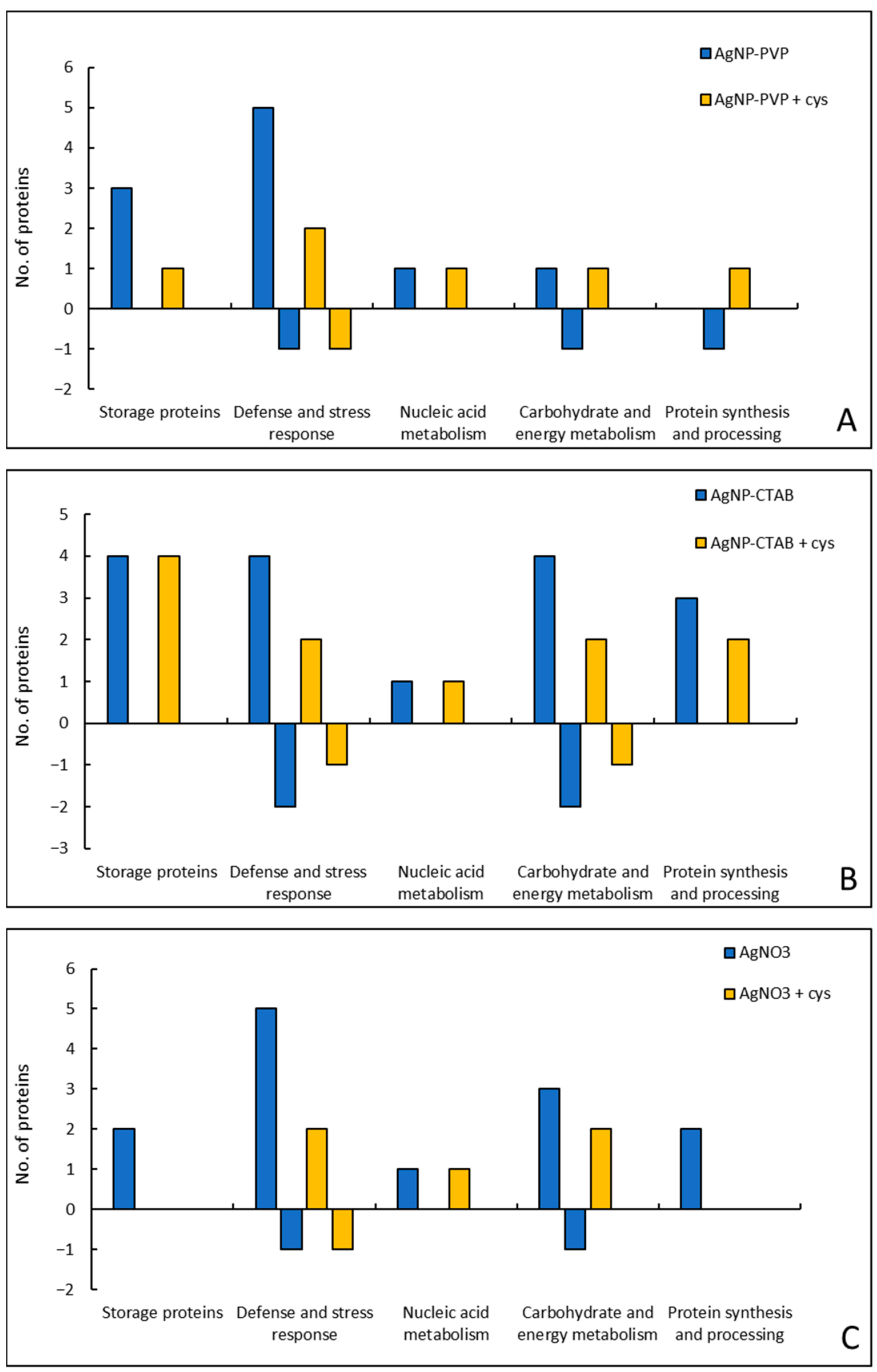
Analysis of responsiveness of differentially abundant proteins, according to their functional categorization, in tobacco seedlings 7 days after exposure to 100 μM AgNP-PVP (**A**), AgNP-CTAB (**B**), and AgNO_3_ (**C**), alone and in combination with 500 μM cysteine (cys). Positive axis of the ordinate shows the number of up-regulated proteins, and the negative axis shows the number of down-regulated proteins compared to the control.

Only eight of the identified proteins showed equal response upon treatment with AgNP-PVP, AgNP-CTAB and AgNO_3_ (Table 2, Figure 7). The greatest overlap in the differential abundance of proteins was observed between AgNP-CTAB and AgNO_3_ treatments (14 proteins in total). Between two types of AgNPs, changes in the abundance of 11 different proteins were detected, while in AgNP-PVP and AgNO_3_ treatments similar response was observed in 10 proteins. The addition of cysteine had a significant impact on reducing AgNP-PVP and AgNO_3_ effects; changes in the abundance of almost 60% of proteins were mitigated in the combined treatments. On the other hand, cysteine addition had a somewhat weaker impact on AgNP-CTAB in combined treatments, alleviating the effects in only 35% of the detected proteins. 

**Figure 8:**
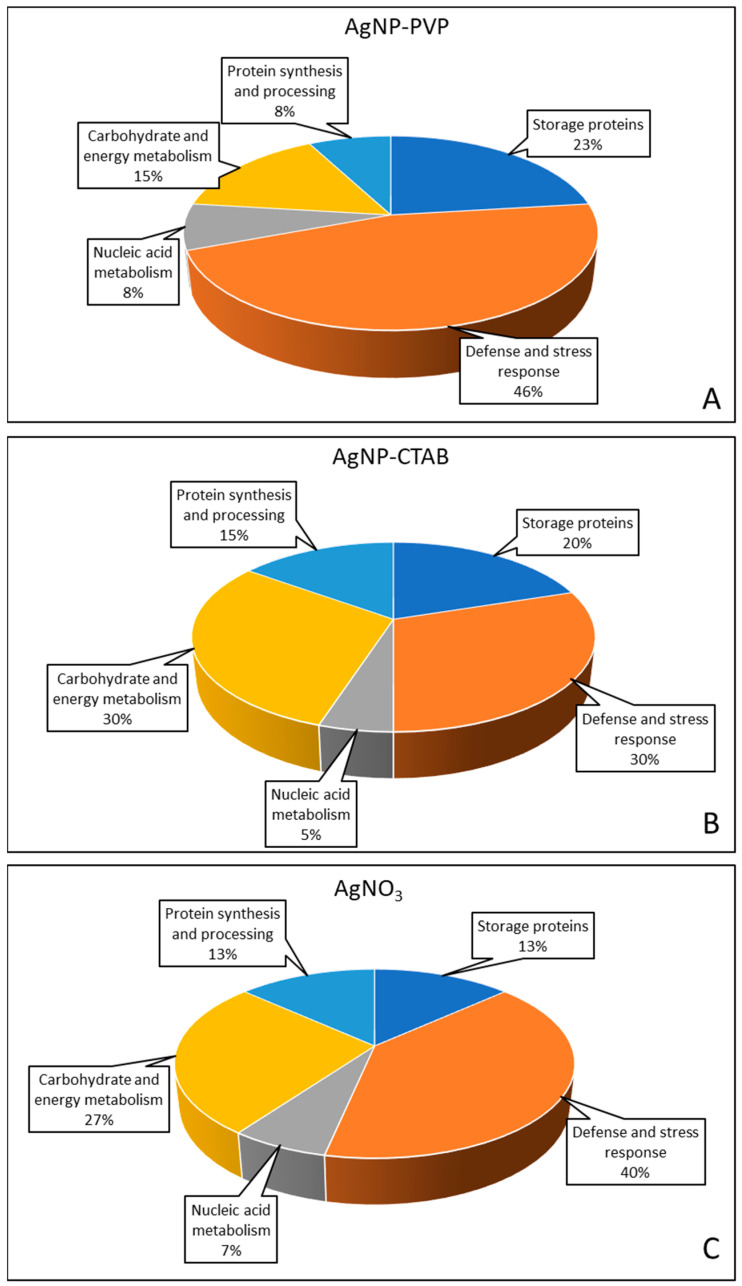
Functional categorization of the differentially abundant proteins in tobacco seedlings seven days after exposure to 100 μM AgNP-PVP (**A**), AgNP-CTAB (**B**) and AgNO_3_ (**C**).

Identified proteins were described according to their localization, molecular and biological function and were divided into five functional categories (Table 2, Figure 8). The highest number of proteins involved in AgNP-PVP response belonged to the defense and stress response (46%) category, followed by storage proteins (23%) and carbohydrate and energy metabolism (15%) categories, while the smallest number of proteins belonged to categories of nucleic acid metabolism and protein synthesis and processing (both with 8%). The highest number of differently abundant proteins after AgNP-CTAB treatment belonged to the categories of defense and stress response and carbohydrate and energy metabolism (both 30%), followed by categories of storage proteins (20%), protein synthesis and processing (15%) and finally the category of nucleic acid metabolism (5%). Similarly to AgNP-PVP, AgNO_3_ treatment predominantly caused changes in the abundance of proteins from the category of defense and stress response (40%). The following categories were carbohydrate and energy metabolism (27%), protein synthesis and processing (13%), storage proteins (13%) and nucleic acid metabolism (7%).

## 3. Discussion

The impact of the physico-chemical characteristics of AgNPs on their stability and subsequent biological interactions has been confirmed in multiple studies, with special emphasis placed on the size of AgNPs as the deciding factor of their phytotoxicity [38,39,40,41]. However, our study has shown that different coatings used for AgNP stabilization should also be considered in the assessment of their toxicity. Namely, coatings used during synthesis define the shape and, even more importantly, the charge of AgNPs [17]. In the plant studies, it was shown that, compared to AgNP-PVP which bear a slight negative charge, positively charged AgNP-CTAB can exhibit a strong toxic effect which can be attributed to their possible binding to the negatively charged plant cell wall [23,38], which corroborates our results. The use of other positively charged coatings, such as cystamine [42] or DDAB [21], also resulted in more severe plant responses compared to the effects of AgNPs with negative or uncharged coatings. The physico-chemical characteristics of AgNPs, defined by the coatings applied for their stabilization, significantly affect their behavior in the environment [43,44,45]. The differences in stability of AgNP-PVP and AgNP-CTAB in the nutrient medium obtained in this study strongly suggest that AgNP stability in the certain biological conditions also plays an important role in their toxicity. Namely, AgNP-CTAB were prone to fast aggregation probably induced by the loss of the coating from their surface, as shown by the rapid change in ζ potential. On the other hand, AgNP-PVP showed better stability in the nutrient medium alone; however, the addition of cysteine led to the faster dissolution of Ag^+^ ions from their surface, probably because sulfhydryl-containing organic compounds, such as thiols, can decrease aggregation rates of AgNPs by adsorbing to their surface, changing their surface charge, which results in increased Ag^+^ dissolution [37,46]. Overall, steric repulsion of PVP coating provided higher colloidal stability for AgNPs in a ½ MS medium compared to the use of electrostatic stabilization with CTAB. 

The induction of oxidative stress is considered to be one of the most important mechanisms of AgNP phytotoxicity [13,36]. Many authors attribute the increased formation of cellular ROS to the oxidative dissolution of Ag^+^ ions from the AgNP surface [38,47,48,49]. In the present study, the accumulation of silver was alike at all corresponding concentrations of PVP and CTAB-coated AgNPs, as well as AgNO_3_, but AgNP-PVP and AgNP-CTAB showed higher potential for ROS production compared to AgNO_3_ treatments. As AgNP uptake in root cells was confirmed by TEM-EDX, it suggests that the toxic effects on tobacco seedlings were at least partially due to AgNP nanoparticulate form. However, cysteine addition alleviated the effects of AgNP-PVP and AgNO_3_, but failed to reduce AgNP-CTAB toxicity. On top of that, only AgNP-CTAB treatment resulted in increased H_2_O_2_ content in tobacco seedlings. Moreover, treatment with CTAB coating alone led to increased ROS, as well as H_2_O_2_ values. This was further corroborated by the increased values of MDA, protein carbonyls and % tDNA measured in tobacco seedlings after the highest tested concentration of AgNP-CTAB, as well as CTAB coating alone. These findings suggest that coating itself can play an important part in the induction of oxidative stress. Considering that in our research the CTAB coating alone also exerted high toxicity towards lipids, proteins and DNA molecules, it is important to acknowledge its role in AgNP-CTAB toxicity. Similarly, gold nanorods (AuNR) stabilized with CTAB also exhibited high genotoxic effects on onion roots, which were 40× higher compared to AuNR-PEG of the same concentration, and what is more, CTAB coating alone also showed strong toxic effects, which were not observed in treatments with PEG coating [50]. 

The oxidative damage of biomolecules, which occurs after ROS production exceeds the capacity of antioxidant defense system, has also been recorded upon the exposure of tobacco seedlings to citrate-coated AgNPs and AgNO_3_ [51], as well as to cadmium [52,53]; thus, oxidative damage can be generally used as an indicator of metal-imposed oxidative stress in this plant species. Higher concentrations of MDA after AgNP-CTAB compared to AgNP-PVP treatment have also been measured in onion roots [38]. The same research also reported a significant increase in protein carbonyl content after both AgNP treatments compared to the control, and a higher increase in % tDNA in AgNP-PVP treatment compared to AgNP-CTAB, which can suggest that onion as species is more sensitive to AgNPs. However, the size of the applied AgNP-PVP and AgNP-CTAB was significantly smaller in the aforementioned research (9.4 and 5.6 nm, respectively), which significantly amplified both AgNP-PVP and AgNP-CTAB toxicity. Moreover, the use of ultrapure water as a medium for onion root growth surely contributed to better stability in both AgNPs during the treatment, possibly reducing the effect of the CTAB coating alone and contributing to higher AgNP-PVP toxicity overall. Interestingly, the response to AgNP-imposed stress seems to be dependent on the plant developmental stage as well. Namely, when adult tobacco plants were exposed to AgNP-PVP and AgNP-CTAB as well as AgNO_3_, a higher Ag uptake, and consequently a higher impact on oxidative stress parameters, was obtained in treatments with both types of nanosilver compared to ionic silver, although no severe damage to important biomolecules was observed [24]. Similar differences between seedling- and adult-plant stages of tobacco were also reported after exposure to cadmium, zinc and copper [52,53]. To protect themselves against imposed oxidative damage, plants have developed efficient machinery for the neutralization of ROS and maintenance of cell integrity and function, comprising various enzymatic and nonenzymatic components [4,17]. The activity of antioxidant enzymes was mostly unaffected by AgNP-PVP and AgNO_3_, which is in accordance with the absence of oxidative damage recorded after those treatments. However, a higher abundance of APX and glutathione S-transferase (GST), shown after the proteomic analysis, indicates the disturbance of oxidative balance upon treatments with AgNP-PVP and AgNO_3_. AgNP-CTAB, on the other hand, significantly increased the activity of CAT and APX, and decreased activity of PPX at the highest tested concentration, which, combined with higher abundance of APX and GST as well, further confirms their high toxicity and is in agreement with the increased H_2_O_2_ concentrations that were measured. The addition of cysteine only partially alleviated AgNP-CTAB-induced alterations in the activity of enzymes in our research, showing that Ag^+^ ions play only a smaller role in their toxicity. Elevated CAT and APX activities were also recorded upon the exposure of tobacco seedlings to AgNP-citrate and AgNO_3_ [51] as well as to cadmium, zinc and copper [52,53], which suggests that the activation of antioxidant enzymes that mitigate oxidative stress by converting cellular H_2_O_2_ to H_2_O and O_2_ is the main mechanism to cope with metal-imposed oxidative stress in seedling-stage tobacco. However, a study performed on adult tobacco plants upon exposure to both PVP- and CTAB-coated AgNPs revealed a strong activation of SOD, scavenger of superoxide anion radicals [24]. Complementary results were also obtained for tobacco seedlings and adult plants after exposure to cadmium, zinc and copper [52,53]. These findings imply that the activation of mechanisms involved in the protection against nanosilver and/or metal stress might be determined by the phase of development of the particular plant species. 

Even though all applied Ag forms at the highest concentration increased the content of proline and GSH/GSSG ratio in tobacco seedlings, those effects were most pronounced after AgNP-PVP treatments, showing that nonenzymatic antioxidants play a more important role in the detoxification of this treatment compared to enzymatic antioxidants. The measured increase in proline and glutathione content after AgNP-PVP and AgNO_3_ treatments was mostly abolished in the combined treatments with cysteine, proving Ag^+^ ions to be the main source of AgNP-PVP toxicity. A significant increase in proline, and particularly glutathione content, was also recorded in adult tobacco plants exposed to differently coated AgNPs and AgNO_3_ [24], thus confirming an important role of nonenzymatic antioxidants in the detoxification process in tobacco. Higher values of proline were also reported after AgNP treatments in *Lupinus termis* [54], *Oryza sativa* [55] and *Spirodela polyrhiza* [56], and difference between the effects of positively charged AgNP-cystamine and negatively charged AgNP-citrate were also observed in *Triticum aestivum* experiments [42], confirming the results obtained in this research. Several researchers have also shown increased values of GSH upon different AgNP treatments [39,57]. However, measuring the ratio of GSH and GSSG, as was carried out in our research, can give a better insight into the redox state of the cell. Namely, even though first part of the cell response to oxidative stress involves the decrease in the GSH/GSSG ratio due to enhanced synthesis of GSSG, longer periods of stress lead to enhanced antioxidant activity and increased capacity of glutathione-ascorbate cycle, which ultimately results in overcompensation and increased GSH/GSSG ratio [58,59]. 

Proteomic analyses revealed 26 proteins with altered abundance after treatments with AgNP-PVP, AgNP-CTAB and AgNO_3_ and their combination with cysteine. Most of the detected proteins were up-regulated in the treatments compared to the control, and only a small number was down-regulated. The highest number of differently abundant proteins was detected after AgNP-CTAB treatment, followed by AgNO_3_ treatment, and finally AgNP-PVP treatment. Cysteine addition significantly reduced the effects of AgNP-PVP and AgNO_3_, but showed less effect in combined treatments with AgNP-CTAB.

The largest number of differentially abundant proteins in Ag treatments belonged to the group defense and stress response, where the majority of detected proteins were involved in response to oxidative stress. Up-regulation of APX and GST was detected after all types of Ag treatments, as mentioned before. AgNP-PVP and AgNO_3_, both alone and in combination with cysteine, induced up-regulation of the 70 kDa heat shock protein (Hsp 70), while up-regulation of Hsp 70–Hsp 90 organizing protein 2 was also found after AgNP-PVP treatment. Both proteins are involved in the maintenance of protein homeostasis, which is highly prone to change during stress conditions such as heat, drought or high salinity [60]. A higher abundance of proteins from this group was already reported after the exposure of tobacco seedlings [51], as well as *Eruca sativa* plants [26] to citrate-coated AgNPs. Studies in which changes in proteome expression were analyzed in tobacco seedlings [51] and adult plants [61] after exposure to AgNP-citrate have shown that pathogenesis-related (PR) proteins are involved in the response to AgNP-induced stress in this plant species. These findings are also confirmed by results obtained in this research, where the down-regulation of pathogenesis-related protein 1C-like, after treatments with AgNP-PVP, AgNP-CTAB and AgNO_3_, as well as their combination with cysteine, have been recorded. This protein group is usually involved in the response to wounding or plant infections, but can also participate in the response to heavy metal stress [62,63].

The second biggest group affected by AgNP treatments was carbohydrate and energy metabolism. The effect of different forms of Ag was especially visible in the differential abundance of proteins involved in photosynthesis, the most important physiological process in plants [64]. AgNP-CTAB and AgNO_3_ treatments resulted in the down-regulation of large chain of ribulose biphosphate carboxylase/oxygenase (Rubisco). Moreover, down-regulation of Rubisco activase 2, important for Rubisco activation [64], was measured after AgNP-CTAB and AgNP-PVP treatment. Changes in the expression of Rubisco activase 2 were also recorded in tobacco seedlings [51] and the leaves of adult plants after their exposure to citrate-coated AgNPs and AgNO_3_ [61], which indicates that Rubisco and its activase are proteins which are particularly sensitive to Ag-imposed stress in tobacco. Rubisco represents a key enzyme responsible for CO_2_ fixation onto ribulose-1,5-biphosphate (RuBP) during the first part of the Calvin cycle. The stress-induced inhibition of photosynthesis decreases the rate of electron transport and ATP synthesis, disabling proper regeneration of RuBP and limiting CO_2_ fixation [64,65,66]. Down-regulation of Rubisco after AgNP treatments was also reported in *Glycine max* [67]. Both AgNP-CTAB and AgNO_3_ treatments induced up-regulation of light-harvesting chlorophyll a/b binding proteins (LHCB), which play an important role in plant adaptation to environmental stress [68,69,70]. These findings, combined with the previously reported down-regulation of oxygen-evolving enhancer protein 2 (OEE2) [67] and photosystem II stability/assembly factor (HCF136) [71], all integral parts of PSII [70,72], indicate a significant damage of the photosynthetic apparatus and inhibition of photosynthesis upon AgNP treatments. The response of tobacco seedlings to AgNP-induced stress was also visible in the differential abundance of glycolytic proteins. Up-regulation of phosphopyruvate hydratase, the enzyme responsible for the catalysis of 2-phosphoglycerate into phosphoenolpyruvate [73], a main pyruvate precursor, was detected after AgNP-CTAB and AgNO_3_ treatments. Up-regulation of plastid aldolase, glyceraldehyde 3-phosphate dehydrogenase (GAPDH) and triose-phosphate isomerase (TPI), important enzymes of glycolytic cycle, was detected in tobacco seedlings upon treatment with AgNP-citrate, which suggests that a higher abundance of glycolytic proteins is important to provide sufficient energy for the young tobacco plants during stress periods [51]. However, the exposure of adult tobacco plants to AgNP-citrate showed contrary results and caused the down-regulation of plastid aldolase, GAPDH and TPI in leaves [61], suggesting that tobacco plants employ different mechanisms to cope with AgNP-imposed stress depending on their developmental status. GAPDH was also down-regulated in rice [74] and wheat [75] seedlings, showing that abundance of glycolytic proteins upon AgNP treatments also depends on plant species. Higher demand for energy upon AgNP–induced stress was also manifested through the up-regulation of dihydrolipoyllysine-residue succinyltransferase, a protein involved in plant TCA cycle, which was observed in all treatments, as well as the up-regulation of 3-ketoacyl-CoA thiolase 2, an enzyme involved in fatty acid beta-oxidation, after AgNP-CTAB treatment. 

Significant changes in the abundance of storage proteins were also detected. Both AgNP-PVP and AgNP-CTAB, as well as AgNO_3_, induced up-regulation of vicilin-like antimicrobial peptides 2-3 and 11S globulins, while only AgNP treatments increased the abundance of legumin A. Storage proteins have an important function as nutrient reservoirs during germination stages, but can also be found in other plant developmental stages [76]. Although they usually serve as protein storage during plant growth and development, their increased abundance was also observed in the response against virus infection [77,78]. However, to our knowledge, this is the first time that storage proteins were involved in plant response to Ag-induced stress. 

An important group of proteins for the maintenance of cell homeostasis under stress conditions is protein synthesis and processing. Up-regulation of protein disulfide-isomerase, involved in protein folding processes, was detected after AgNP-CTAB and AgNO_3_ treatments. Moreover, AgNP-CTAB also induced up-regulation of chaperonin 60 subunit beta 2 that holds the same cellular function. Enhanced abundance of proteins in this group was also reported for tobacco seedlings treated with citrate-coated AgNPs [51], as well as in the research of AgNP effects on seedlings of wheat [71] and rice [74], which corroborates the importance of proper protein folding processes for response of young plants to Ag-induced stress. Accordingly, the catabolism of the damaged proteins also has an important role following AgNP treatments. The up-regulation of proteasome subunit alpha type detected after AgNP-CTAB and AgNO_3_ treatments in this research, as well as up-regulation of other proteins involved in protein degradation processes caused by their irreversible oxidative damage [75,79], attest to the significant protein damage caused by AgNPs.

Only one protein from the group of nucleic acid metabolism exhibited enhanced abundance after treatments with AgNP-PVP, AgNP-CTAB and AgNO_3_. This was S-adenosylmethionine (SAM) synthase, a protein involved in the epigenetic regulation of plants through the transfer of methyl groups. The methylation of DNA, RNA, proteins, lignins and flavonoids plays an important role in regulation of plant development and response to biotic and abiotic stress [80,81,82], suggesting an important role of SAM synthase in response to AgNP-induced stress.

## 4. Materials and Methods

### 4.1. Materials

NaOCl was purchased from Kemika (Zagreb, Croatia). For centrifugal ultrafiltration, Amicon Ultra-4 3K devices (Merck Millipore, Burlington, MA, USA) were employed. Ultrapure water was ion-free Milli-Q water with 18.2 MΩ-cm resistivity, also from Merck Millipore. For AgNP soluiton dialysis, 10 kDa molecular weight cut-off (MWCO) membrane tubing from Thermo Scientific (Waltham, MA, USA) was used. The GelStarTM Nucleic Acid Gel Stain was obtained from Lonza (Basel, Switzerland). For protein separation by isoelectric focusing (IEF), dry immobilized pH gradient (IPG) strips (Immobiline DryStrip, 13 cm, pH 3–10 NL) from GE Healthcare (Chicago, IL, USA) were employed. Other reagents and chemicals applied in this research were obtained from Sigma-Aldrich (St. Louis, MO, USA).

### 4.2. AgNP Synthesis and Characterization

**Figure 9:**
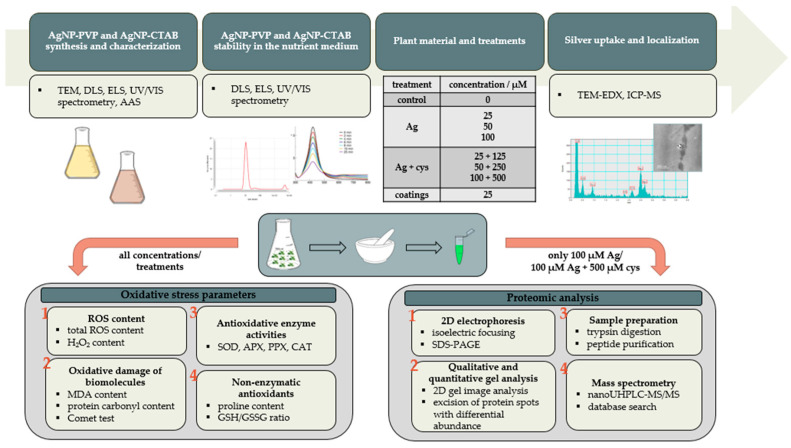
Schematic representation of the experimental setup. Ag—all silver forms (AgNP-PVP, AgNP-CTAB, AgNO_3_), cys—cysteine.

Detailed descriptions of AgNP-PVP and AgNP-CTAB synthesis, as well as methods for their physico-chemical characterization, can be found in our previous publication [22]. Briefly, for the preparation of AgNP-PVP, 0.02 g of AgNO_3_ was dissolved in 120 mL of ultrapure water with the addition of 0.019 g of PVP (average molecular weight of 40,000 g·mol^−1^). The solution was brought to boiling and was rapidly supplemented with 5 mL of 1% aqueous sodium citrate solution, after which it was boiled again. Upon color alteration from transparent to light yellow, solution was cooled to room temperature and dialyzed against ultrapure water in a 10 kDa MWCO membrane tubing for 24 h. For AgNP-CTAB synthesis, 0.02 g of AgNO_3_ was mixed with 0.0043 g of CTAB coating and dissolved in 62.5 mL of ultrapure water. Obtained solution was cooled and supplemented with 0.01 g of ascorbic acid by burette in a constant stream with continuous stirring, upon which solution changed color from light yellow to orange and was dialyzed against ultrapure water in a membrane tubing of 10 kDa MWCO for 24 h. Synthesis of both AgNP types was confirmed with UV-Vis spectroscopy (Unicam, Cheshire, UK), considering the presence and position of surface plasmon resonance (SPR) peak (Figure 9). AgNPs’ visualization (4 replicas for each AgNP type) was achieved by employment of monochromated TF20 (FEI Tecnai G2, Hillsboro, OR, USA) transmission electron microscope (TEM) equipped with energy-dispersive X-ray spectroscopy (EDX). AgNPs’ size and charge were determined with dynamic light scattering (DLS) and electrophoretic light scattering (ELS), respectively, using Zetasizer Nano ZS (Malvern Panalytical, Malvern, UK). DLS results are presented as volume distributions and represent mean value ± SD of 10 measurements. ζ potentials are reported as mean value ± SD of 5 measurements. Concentration of Ag in AgNP suspensions, used for subsequent preparation of working solutions, was measured using ELAN DRC-e inductively coupled plasma mass spectrometer (ICP-MS) (Perkin Elmer, Waltham, MA, USA) in 5 replicas. The same instrument was used to assess dissolution of silver from AgNP-PVP and AgNP-CTAB after centrifugal ultrafiltration (Amicon Ultra-4 3K).

### 4.3. Plant Material and Treatments

For cultivation and treatment of tobacco (*Nicotiana tabacum* L.) seedlings in in vitro conditions, liquid half-strength nutrient medium of Murashige and Skoog (½ MS) [83] was used. Tobacco seeds were surface-sterilized with 50% (*v*/*v*) NaOCl, as described in Biba et al. [22], and put in sterilized Erlenmeyer flasks (100 mL), which contained 5 mL of liquid ½ MS medium. They were left to germinate and grow on a shaker, with periodical supplementation with fresh nutrient medium. After 3 weeks, the existing nutrient medium was removed and subsequently replaced with ½ MS medium containing AgNP-PVP or AgNP-CTAB, alone or in combination with cysteine (applied in a cysteine:silver molar ratio 5:1), to obtain 25, 50, and 100 µM concentrations of Ag and 125, 250, and 500 µM concentrations of cysteine, respectively. Even though the AgNP-induced phytotoxicity mechanism is not completely clarified, it is a prevailing belief that the AgNP toxicity is directed by a release of Ag^+^ ions (reviewed in Biba et al. [27]). Therefore, the same concentrations of AgNO_3_, as a source of Ag^+^ ions, were also included in the study, either alone or in combination with cysteine. The effects of 25 µM concentration of each coating (PVP or CTAB) were also evaluated. Tobacco seedlings were exposed to the abovementioned treatments for 7 days in the growth chamber at 24 ± 1 °C and 90 µmol m^−2^ s^−1^ light intensity, with 16/8 h light/dark cycles. The experiment was performed 2× with 6 replicas for each treatment.

### 4.4. AgNP Stability in Liquid ½ MS Medium

Stability of 100 μM AgNP-PVP and AgNP-CTAB in a liquid ½ strength MS medium, alone or in combination with 500 μM cysteine, was evaluated using UV-Vis spectroscopy, as described in Peharec Štefanić et al. [23]. Changes in position and intensity of SPR peak of prepared solutions, kept in the same conditions as the plant material, were monitored at regular time intervals during the period of 7 days. To better understand initial transformations of AgNPs in the nutrient medium, changes in hydrodynamic diameter (DLS) and ζ potential (ELS) were monitored for the first 4 h after the addition of AgNPs [23]. DLS results, presented as volume distributions, represent mean value ± SD of 10 measurements. ζ potentials are reported as mean value ± SD of 5 measurements.

### 4.5. Silver Uptake and Localization

For Ag uptake measurements, tobacco seedlings treated with either AgNPs or AgNO_3_ were firstly washed with ultrapure water, after which they were oven-dried for 24 h at 80 °C until a constant weight was obtained. Samples were prepared for analysis according to the modified US EPA method 3052 as previously described [22,24] and silver content was measured using ELAN DRC-e ICP-MS. Calibration curve achieved with standards of specific concentrations was applied in order to calculate the Ag concentration. The limit of quantification (LOQ) and detection limit were 0.10 and 0.05 mg kg^−1^, respectively. For seedlings exposed to AgNO_3_, spike recovery tests were 93.5%, while for those exposed to AgNPs were 91.8% (AgNP-PVP) and 94.5% (AgNP-CTAB). 

Seedling tissue sections used for AgNP-PVP and AgNP-CTAB localization, upon exposure to 100 µM concentrations of both types of AgNPs, were prepared as reported in our previous studies [23,38]. Fixation was performed in a 1% (*w*/*v*) glutaraldehyde in 50 mM cacodylate buffer of pH 7.2 (1 h at +4 °C), after which the sections were washed 2× in a pre-cooled 50 mM cacodylate buffer of pH 7.2. Post-fixation was performed in a 1% (*w*/*v*) osmium tetroxide in the same buffer (1 h at +4 °C), after which sections were washed for 10 min in an ice-cold water. For dehydration, tissue sections were incubated in a graded series of ethanol and subsequently embedded in Spurr’s resin. 2% (*w*/*v*) uranyl acetate and 2% (*w*/*v*) lead citrate were used for staining of ultrathin sections. Monochromated TF20 TEM was applied for uptake confirmation of either AgNP-PVP or AgNP-CTAB in the tobacco cells.

### 4.6. Protein Extraction

Proteins from seedlings were extracted as previously described in Peharec Štefanić et al. [51]. Briefly, 0.6 g of lyophilized tissue was ground in 1.5 mL of 100 mM potassium phosphate buffer, pH 7.0, using a chilled mortar and pestle, with the addition of 50 mg of insoluble PVP. Obtained homogenates were centrifuged for 15 min at 20,000× *g* and 4 °C, and the supernatants were transferred to clean tubes and centrifuged again for 60 min under the same conditions. Protein concentration was determined according to Bradford [84] assay, using bovine serum albumin (BSA) as a standard. Obtained extracts were used for measurement of total ROS content, quantification of protein carbonyls and enzymatic activities assays.

### 4.7. ROS Content 

Total ROS content was determined using fluorescent probe dihydroethidium (DHE) that detects mainly superoxide radical [85]. For measurement, 50 μL of 20 μM DHE was added to 50 μL of the extract in a 96-well plate. Fluorescence was read on a microplate reader (Infinite 200 PRO NanoQuant, Tecan, Zürich, Switzerland) without further incubation, with excitation set at 520 nm and emission at 600 nm. Obtained results are expressed as percentage of control sample.

Hydrogen peroxide (H_2_O_2_) content was measured as described in Alexieva et al. [86]. Samples were prepared by grinding 150 mg of fresh seedling tissue in 2 mL of cold 0.1% (*w*/*v*) trichloroacetic acid (TCA) with the addition of 50 mg of PVP and liquid N_2_. After centrifugation (10 min at 20,000× *g* and 0 °C), 0.5 mL of obtained supernatants was mixed with 0.5 mL of 10 mM potassium-phosphate buffer, pH 7.0, and 1 mL of 1 M potassium iodide (KI). Absorbance was read at 390 nm, and H_2_O_2_ concentration in the samples was calculated using a calibration curve obtained with H_2_O_2_ solutions of known concentrations. Results are expressed as μmol g^−1^ of fresh weight. 

### 4.8. MDA and Protein Carbonyl Content

Modified method by Heath and Packer [87] was implemented to determine lipid peroxidation level. 60 mg of lyophilized tissue was extracted in 1.5 mL of a reaction mixture consisting of 0.3% (*w*/*v*) thiobarbituric acid (TBA) and 10% (*w*/*v*) TCA, using a mortar and pestle. After 30 min of incubation at 95 °C, homogenates were cooled and centrifuged for 15 min at 20,000× *g* and 4 °C. Malondialdehyde (MDA) content in the obtained supernatants was measured at 532 nm with the subtraction of the value measured at 600 nm to correct for nonspecific turbidity, and calculations were performed using the MDA molar absorption coefficient (155 mM^−1^ cm^−1^). Results are expressed as μmol mg^−1^ of dry weight.

Protein carbonyl content was determined using the method by Levine et al. [88]. 200 μL of protein extracts (Section 4.6) was combined with 300 μL of 10 mM 2,4-dinitrophenylhydrazine (DNPH) dissolved in 2 M HCl and incubated in dark at room temperature for 60 min. To precipitate the proteins, 500 μL of 10% (*w/v*) TCA was added to each sample. After 10 min incubation at −20 °C, samples were centrifuged (10 min at 20,000× *g* and 4 °C), supernatants were removed and pellets were washed three times with 500 μL solution of ethanol/ethylacetate (1:1, *v*/*v*) to remove excess reagent. Pellets were then dissolved in 1 mL of 6 M urea dissolved in 20 mM potassium phosphate buffer (pH 2.4), and absorbance was read at 370 nm against the reference prepared for each sample in a reaction mixture devoid of DNPH. To estimate protein recovery, absorbance of each sample was also measured at 280 nm. Concentration of protein carbonyls was calculated using a molar absorption coefficient for aliphatic hydrazones of 22 mM^−1^ cm^−1^ and expressed as μmol mg^−1^ of proteins.

### 4.9. Comet Assay

Comet assay was performed as described in Cvjetko et al. [38]. After the mechanical isolation of nuclei in 400 mM Tris-HCl (pH 7.5) at 4 °C, 50 μL of nuclei suspension was mixed with 50 μL of 1% (*w*/*v*) low melting agarose and placed onto a slide. After 10-min denaturation and following 20-min electrophoresis at 26 V and 300 mA in a buffer consisting of 1 mM Na_2_EDTA and 300 mM NaOH, pH 13, slides were neutralized with 400 mM Tris-HCl (pH 7.5), air-dried and stained with 70 μL of GelStarTM Nucleic Acid Gel Stain. A computerized image analysis system (Komet ver. 5, Kinetic Imaging Ltd. Liverpool, UK) was employed to measure tail DNA percentage (% tDNA), a primary measure of DNA damage.

### 4.10. Antioxidative Enzyme Activities

SOD (EC 1.15.1.1) activity was determined according to Beauchamp and Fridovich [89] method based on the inhibition of nitroblue tetrazolium (NBT) reduction. To determine the maximum absorbance, reaction mixture consisting of 13 mM of methionine, 75 μM of NBT, 0.1 M of EDTA and 2 mM of riboflavin was incubated for 8 min in a 15 W light box, and formazan produced by NBT photo reduction was measured at 560 nm. Volume of protein extract (Section 4.6) needed for 50% inhibition of the NBT reduction rate, as defined by one unit (U) of SOD activity, was then added to the reaction mixture and measured as explained previously. Specific activity of SOD was expressed as U mg^−1^ proteins.

PPX (EC 1.11.1.7) and APX (EC 1.11.1.11) activities were assayed according to Nakano and Asada [90]. For PPX measurement, 20 μL of the protein extract (Section 4.6) was added to 980 μL of the reaction mixture (50 mM potassium phosphate buffer, pH 7.0, 20 mM pyrogallol and 1 mM H_2_O_2_). Increase in absorbance at 430 nm, caused by oxidation of pyrogallol (ε = 2.6 mM^−1^ cm^−1^), was measured and PPX specific activity was expressed as μmol purpurogallin min^−1^ mg^−1^ proteins. For APX activity measurement, 180 μL of the protein extract (Section 4.6) was combined with 800 μL of reaction mixture (50 mM potassium phosphate buffer, pH 7.0, and 10 mM EDTA), 10 μL of 0.1 mM ascorbic acid and 10 μL of 12 mM H_2_O_2_. Decrease in absorbance, caused by ascorbate (ε = 2.8 mM^−1^ cm^−1^) consumption, was measured at 290 nm, and specific APX activity was expressed as μmol oxidized ascorbate min^−1^ mg^−1^ proteins.

CAT (EC 1.11.1.6) activity was monitored according to Aebi [91]. For the measurement, 30 μL of the protein extract (Section 4.6) was added to 970 μL of the reaction mixture consisting of 50 mM potassium phosphate buffer (pH 7.0) and 10 mM H_2_O_2_. Decrease in absorbance, indicating H_2_O_2_ (ε = 36 mM^−1^ cm^−1^) decomposition, was recorded at 240 nm. Specific CAT activity was expressed as μmol decomposed H_2_O_2_ min^−1^ mg^−1^ proteins.

### 4.11. Non-Enzymatic Antioxidants 

For determination of proline and glutathione content, 150 mg of the fresh seedlings tissue was extracted in 1.5 mL of 3% 5-sulfosalicylic acid (SA) with the addition of 10 mg of PVP. Homogenates were centrifuged for 15 min at 10,000× *g* and 4 °C, and supernatants were stored on ice until use.

Proline content was measured according to Bates et al. [92]. 500 μL of supernatant was mixed with 500 μL of acid ninhydrin solution and 500 μL of glacial acetic acid, and incubated at 95 °C for 60 min. After cooling, mixture was extracted in 1.2 mL of toluene, and absorbance of chromophore containing toluene layer was read at 520 nm. Concentration of proline was determined using the calibration curve and expressed as μmol proline per gram of fresh weight.

For determination of total glutathione content, a method by Salbitani et al. [93] was applied. Reduced glutathione (GSH) was measured at 412 nm after adding 100 μL of the supernatant to 750 μL of the reaction mixture [1.5 mg mL^−1^ 5,5 -dithio-bis-(2-nitrobenzoic acid (DTNB), 0.1 M potassium phosphate buffer (pH 7,0) and 1 mM EDTA] and 20 min incubation at room temperature. Total glutathione content was then determined using enzymatic recycling method. 15 μL of glutathione reductase (GR, 1 U) and 50 μL of NADPH (0.32 mg mL^−1^) were added to the same reaction mixture, and total glutathione was measured at 412 nm after 5 min incubation at room temperature. GSH and total glutathione content were determined using the calibration curve, and oxidized glutathione (GSSG) was then calculated as the difference between total and reduced glutathione content. Results are expressed as μmol per gram of fresh weight and shown as GSH/GSSG ratio.

### 4.12. Proteomic Analysis

Changes in proteomic profiles of tobacco seedlings treated with 100 μM AgNP-PVP, AgNP-CTAB and AgNO_3_, or their combination with 500 μM cysteine, were analyzed. For protein extraction, a modified phenol method was applied [51,61,94]. Briefly, 150 mg of lyophilized tissue was extracted in 6 mL of the extraction buffer [500 mM Tris, 50 mM EDTA, 700 mM sucrose, 100 mM potassium chloride (KCl), 1 mM phenylmethylsulfonyl fluoride (PMSF), and 2% β-mercaptoethanol], incubated on ice for 10 min and extracted in 6 mL of phenol. Protein precipitation was obtained by applying the ice-cold precipitation solution [0.1 M ammonium acetate (NH_4_CH_3_CO_2_) in methanol] at −20 °C overnight. Pellets were washed with 3 mL of pre-cooled precipitation solution 3× and with 3 mL of ice-cold acetone, after which they were dissolving in 500 μL of isoelectric focusing (IEF) buffer [9 M urea and 4% (*w*/*v*) 3-((3-cholamidopropyl) dimethylammonio)-1-propanesulfonate (CHAPS)], to which 2 mg mL^−1^ of dithiothreitol (DTT) and 5.2 μL of ampholytes were added. Modified Bradford method [95] was applied for determination of protein concentration using the BSA as a standard.

400 μg of proteins of each sample, stained with 5 μL of bromphenol blue, was then loaded on dry immobilized pH gradient (IPG) strip (Immobiline DryStrip, 13 cm, pH 3–10 NL) and left to rehydrate for 16 h at room temperature. Proteins were separated using two-dimensional electrophoresis (2-DE), as previously published in Peharec Štefanić et al. [51,95]. In the 1st dimension, proteins were separated by IEF in the Ettan PGphor 3 system (GE Healthcare) with the program of the following steps: 1 h at 500 V (step and hold), 1 h at 1000 V (gradient), 3 h at 8000 V (gradient), and finally 4 h at 8000 V for (step and hold), with a total run of 30,000 V h. The 2nd dimension of 2-DE was sodium dodecylsulphate-polyacrylamide gel electrophoresis (SDS-PAGE), for which equilibration of IPG strips had to be performed in 2 steps. For the 1st equilibration step, strips were incubated in the equilibration buffer (0.05 M Tris-HCl of pH 8.8, 6 M urea, 2% SDS (*w*/*v*)) supplemented with 130 mM DTT for protein reduction. In the 2nd equilibration step, strips were incubated in the equilibration buffer of the same composition, which instead of DTT contained 135 mM iodoacetamide (IAA) for protein alkylation. Upon equilibration, strips were shortly submerged in an electrode buffer (0.025 M Tris, 0.192 M glycine, 10% (*w*/*v*) SDS, pH 8.3), after which they were mounted on the vertical polyacrylamide slab gel (10% T, 2.67% C, with the addition of 10% SDS). For SDS-PAGE, PROTEAN II xi system (BioRad, Hercules, CA, USA) was used. The 2nd dimension was firstly run for 30 min at 100 V, and then run until the end at 220 V. 

Commassie Brilliant Blue (CBB) R-250 staining solution (0.1% (*w*/*v*) CBB, 45% (*v*/*v*) methanol, and 10% (*v*/*v*) glacial acetic acid) was applied for protein spot visualization. Gels were subsequently scanned (Epson Perfection, V700 Photo, Hillsboro, OR, USA) and analyzed using the ImageMaster 2D Platinum software ver. 7.0 (GE Healthcare, USA), where abundance of each protein spot was represented as volume percentage. The whole experiment was repeated in 3 biological replicates, and protein spots with ± 1.5-fold change showing statistical significance at *p* ≤ 0.05 were considered as differently abundant, excised from the gels and destained in the solution consisting of 10% (*v*/*v*) acetic acid and 40% (*v*/*v*) methanol. Excised gel particles were washed in a solution of 5 mM ammonium bicarbonate (ABC) and 50% (*v*/*v*) acetonitrile 3×, dehydrated in 100% acetonitrile, reduced with 10 mM DTT in 20 mM ABC at 56 °C and alkylated in 55 mM IAA in 20 mM ABC in the dark. Gel particles were then washed 2× in 5 mM ABC and 50% (*v*/*v*) acetonitrile solution, dehydrated in 100% acetonitrile, rehydrated in 50 μL trypsin solution (12.5 ng in 20 mM ABC) and proteins were left to digest overnight at 37 °C. Resulting peptides were extracted in 2 washing steps; 2× in 50% (*v*/*v*) acetonitrile and 1% (*v*/*v*) trifluoroacetic acid (TFA), and 1× in 80% (*v*/*v*) acetonitrile and 1% TFA. All obtained fractions were combined and acetonitrile was evaporated using a vacuum centrifuge (Savant DNA 120 SpeedVac, Thermo Fisher Scientific, Waltham, MA, USA). Extracted peptides were concentrated and purified using C18 StageTips which were washed with methanol and equilibrated with a solution of 2% (*v*/*v*) acetonitrile and 1% (*v*/*v*) formic acid (FA). Peptides were loaded onto equilibrated strips, desalted with 0.1% (*v*/*v*) FA and eluted in 80% (*v*/*v*) acetonitrile and 0.1% (*v*/*v*) FA, and remaining acetonitrile was removed from the samples using the vacuum centrifuge. The digests were analyzed on nanoUHPLC-MS/MS system (EASY-nLC 1200 coupled to Q Exactive Plus mass spectrometer, Thermo Fisher Scientific) after peptide separation with EASY-Spray™ HPLC C18 Column (2 μm particle size, 75 μm × 250 mm). Data analysis was performed using Proteome Discoverer Software ver. 2.4 (Thermo Fisher Scientific). 

### 4.13. Statistical Analysis

STATISTICA 12.0 (Stat Soft Inc., Palo Alto, CA, USA) software package was used for statistical analysis. The obtained data were analyzed using one-way ANOVA followed by post-hoc Duncan test. Differences between means were considered statistically significant at *p* ≤ 0.05.

## 5. Conclusions

AgNP effects on plants are complex and depend on various factors, both environmental and intrinsic. Our results show that Ag uptake and accumulation in tobacco seedlings were similar upon exposure to AgNP- CTAB and AgNP-PVP, as well as AgNO_3_. Moreover, all Ag treatments induced significant ROS formation, thus confirming oxidative stress as the main mechanism of Ag phytotoxicity. However, our study revealed that coatings have an important role in AgNP phytotoxicity; positively charged AgNP-CTAB showed less stability in the exposure medium and exhibited overall more negative effects on oxidative stress appearance and changes in the proteome of exposed tobacco seedlings compared to more stable AgNP-PVP that carry a small negative charge. The highest concentration of AgNP-CTAB treatment, as well as the CTAB coating alone, resulted in increased H_2_O_2_ content and significant damage to lipids, proteins and DNA molecules. Furthermore, differently coated AgNPs induced different responses to oxidative stress in tobacco seedlings; AgNP-CTAB treatment led to a stronger activation of the enzymatic antioxidants, especially CAT and APX, while AgNP-PVP and AgNO_3_ treatments showed greater dependence on the nonenzymatic part of the antioxidant system, significantly increasing proline and GSH content. AgNP-CTAB treatment also induced more pronounced changes in the abundance of proteins involved in the defense and stress response, carbohydrate and energy metabolism and storage proteins compared to AgNP-PVP and AgNO_3_, suggesting an increased need for energy in order to combat the imposed stressful conditions. The addition of cysteine significantly reduced the effects of AgNP-PVP and AgNO_3_ for most of the investigated parameters, indicating that AgNP-PVP toxicity is mainly derived from Ag^+^ ions released from their surface. In contrast, cysteine mostly failed to reduce the effects of AgNP-CTAB, suggesting that they predominantly arise from the intrinsic properties of the nanoparticles themselves, as well as the coating used for their stabilization.

## Figures and Tables

**Table 1:** Content of Ag in tobacco seedlings measured after 7 days of exposure to 25, 50 and 100 µM concentrations of silver nanoparticles coated with either polyvinylpyrrolidone (AgNP-PVP) or cetyltrimethylammonium bromide (AgNP-CTAB) and silver nitrate (AgNO_3_), applied either alone or in combinations with 125, 250 and 500 µM cysteine (cys) in liquid ½ MS medium.

		Ag Content (μg g^−1^ DW)		
Treatment	Concentrations (µM)	AgNP-PVP	AgNP-CTAB	AgNO_3_
Control	0	0 ± 0 ^d^	0 ± 0 ^d^	0 ± 0 ^d^
	25	39.6 ± 1.0 ^c,A^	33.3 ± 6.8 ^c,A^	32.3 ± 6.9 ^c,A^
Ag	50	64.7 ± 6.7 ^b,A^	58.2 ± 4.2 ^b,A^	56.2 ± 4.0 ^b,A^
	100	102.3 ± 7.4 ^a,A^	96.6 ± 3.4 ^a,A^	91.7 ± 1.5 ^a,A^
	25 + 125	24.3 ± 2.6 *^,#^	20.7 ± 1.1 ^#^	9.7 ± 1.8 *^,#^
Ag + cys	50 + 250	42.7 ± 1.5 *^,#^	34.9 ± 7.0 ^#^	17.9 ± 6.1 *^,#^
	100 + 500	81.1 ± 5.4 ^#^	58.0 ± 4.5 *^,#^	50.1 ± 7.6 *^,#^

Data represent the mean values ± standard errors of 2 different experiments with 6 replicates each. Treatments that are significantly different at *p* ≤ 0.05 (one-tailed ANOVA, followed by a Duncan post-hoc test) are labelled with different letters; small letters denote differences between different concentrations of the same treatment type as well as the control; capital letters denote differences between different treatment types of the same concentration; asterisks (*) denote significant differences between treatments with and without cysteine of the corresponding concentration; while hash signs (#) denote significant differences between each treatment with cysteine and the control. The Ag content in the control was below the limit of quantification (LOQ < 0.1 µg g^−1^). DW stands for dry weight.

**Table 2:** List of differentially abundant proteins, their biological and molecular function and cellular localization in tobacco seedlings after the treatment with 100 µM AgNP-PVP, AgNP-CTAB or AgNO_3_ and their combination with 500 µM cysteine. For all treatments with (+cys) or without (−cys) cysteine, ↑ represents higher, ↓ represents lower and = represents equal abundance of protein compared to control (*p* ≤ 0.05, Duncan test).

Protein Spot	Protein Name	MW (kDa)	pI	Molecular Function	Biological Process	Cellular Compartment	Differential Expression		
							AgNP-PVP	AgNP-CTAB	AgNO_3_
***Storage proteins***							**(−cys/+cys)**		
2, 12, 25	legumin A-like	53.6	8.4	nutrient reservoir activity	storage protein	vacuole	**↑/=**	**↑/↑**	**=/=**
9	vicilin-like antimicrobial peptides 2-3	94.6	7.1	nutrient reservoir activity	storage protein	vacuole	**↑/↑**	**↑/↑**	**↑/=**
14, 21	11S globulin subunit beta-like	56.7	8.3	nutrient reservoir activity	storage protein	vacuole	**↑/=**	**↑/↑**	**↑/=**
15, 20, 22	11S globulin seed storage protein 2-like	55.6	7.6	nutrient reservoir activity	storage protein	vacuole	**=/=**	**↑/↑**	**=/=**
***Defense and stress response***									
1	stromal 70 kDa heat shock-related protein, chloroplastic	75.3	5.4	unfolded protein binding	stress response, protein folding	chloroplast	**↑/↑**	**=/=**	**↑/↑**
3	glutathione S-transferase L3-like isoform X2	27.0	4.9	transferase activity	glutathione metabolic process	cytoplasm	**↑/=**	**↑/=**	**↑/=**
18	glutathione S-transferase L3-like isoform X1	27.3	5.1	transferase activity	glutathione metabolic process	cytoplasm	**↑/=**	**↑/↑**	**↑/=**
5	hsp70-Hsp90 organizing protein 2-like	55.5	6.0	Hsp90 protein binding	stress response	cytoplasm, nucleus	**↑/=**	**↓/=**	**=/=**
13	pathogenesis-related protein 1C-like	20.5	5.7	pathogenesis-related protein	plant defense respose	extracellular	**↓/↓**	**↓/↓**	**↓/↓**
16	cytosolic ascorbate peroxidase	27.4	5.7	peroxidase activity	response to oxidative stress	cytoplasm	**↑/↑**	**↑/=**	**↑/↑**
17	L-ascorbate peroxidase 1, cytosolic	27.5	5.9	peroxidase activity	response to oxidative stress	cytoplasm	**=/=**	**↑/↑**	**↑/=**
***Nucleic acid metabolism***									
4	S-adenosylmethionine synthase	42.6	6.1	transferase activity	one-carbon metabolism	cytoplasm	**↑/↑**	**↑/↑**	**↑/↑**
***Carbohydrate and energy metabolism***									
7	phosphopyruvate hydratase	47.8	5.6	lyase	glycolysis	cytoplasm	**=/=**	**↑/↑**	**↑/↑**
8	ribulose bisphosphate carboxylase/oxygenase large chain	52.9	6.9	oxidoreductase	photosynthesis, photorespiration	chloroplast	**=/=**	**↓/=**	**↓/=**
10	3-ketoacyl-CoA thiolase 2, peroxisomal-like	48.8	7.6	acyltransferase	fatty acid beta-oxidation	peroxisome	**=/=**	**↑/↑**	**=/=**
11	ribulose bisphosphate carboxylase/oxygenase activase 2, chloroplastic	48.3	7.7	ATPase activity, Rubisco activator activity	photosynthesis	chloroplast	**↓/=**	**↓/↓**	**=/=**
19	dihydrolipoyllysine-residue succinyltransferase	51.1	8.7	transferase activity	tricarboxylic acid cycle	mitochondrion	**↑/↑**	**↑/=**	**↑/↑**
23	chlorophyll a-b binding protein, chloroplastic	29.3	8.7	chlorophyll binding	photosynthesis	chloroplast	**=/=**	**↑/=**	**↑/=**
***Protein synthesis and processing***									
6	chaperonin 60 subunit beta 2, chloroplastic-like	63.2	5.7	chaperone	protein folding	chloroplast	**=/=**	**↑/↑**	**=/=**
24	proteasome subunit alpha type	26.0	4.8	endopeptidase activity	protein catabolic process	cytoplasm	**=/↑**	**↑/↑**	**↑/=**
26	protein disulfide-isomerase	27.5	8.4	isomerase activity	protein folding	endoplasmic reticulum	**↓/=**	**↑/=**	**↑/=**

## Data Availability

Not applicable.

## Supplementary Materials

The following supporting information can be downloaded at: https://www.mdpi.com/article/10.3390/ijms232415923/s1.
