# Supplementary material for: Effects of Silver Nanoparticles on Physiological and Proteomic Responses of Tobacco (Nicotiana tabacum) Seedlings Are Coating-Dependent
Source: Int J Mol Sci. 2022 Dec 14;23(24):15923. doi: 10.3390/ijms232415923 (PMC9787911; doi:10.3390/ijms232415923)
Supplement: Supplementary file 1 [file ijms-23-15923-s001.zip › Figure S2_AgNP-CTAB stability_revised.pdf]

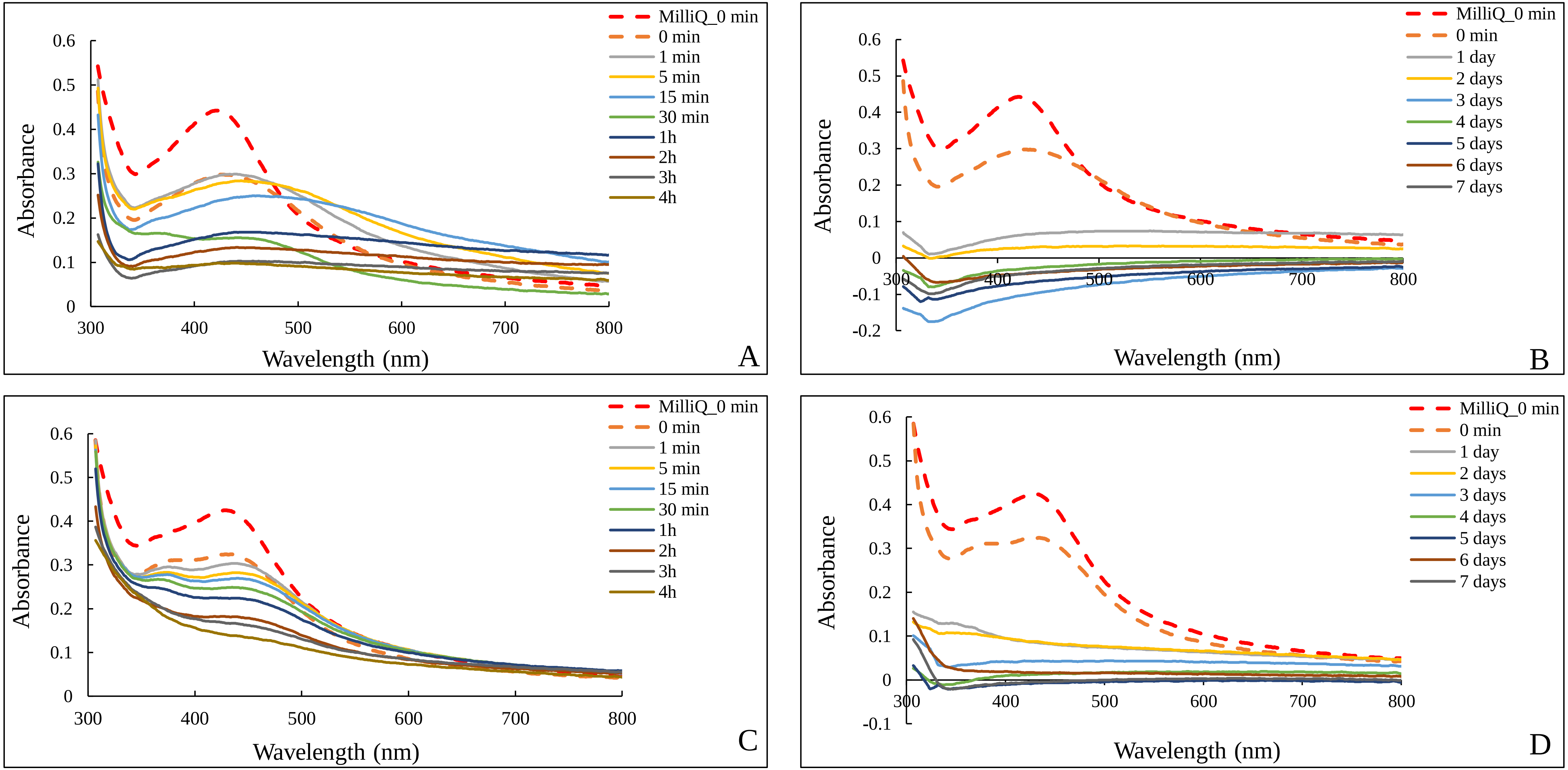

Figure S2. UV-Vis absorption spectra of 100 μM AgNP-CTAB, alone (A, B) or in combination with 500 μM cysteine (C, D), in a liquid ½ strength Murashige and Skoog (MS) medium, recorded over a period of 7 days.
