# Supplementary material for: Effects of Silver Nanoparticles on Physiological and Proteomic Responses of Tobacco (Nicotiana tabacum) Seedlings Are Coating-Dependent
Source: Int J Mol Sci. 2022 Dec 14;23(24):15923. doi: 10.3390/ijms232415923 (PMC9787911; doi:10.3390/ijms232415923)
Supplement: Supplementary file 1 [file ijms-23-15923-s001.zip › Figure S3_DLS temporal analysis_AgNP-PVP.pdf]

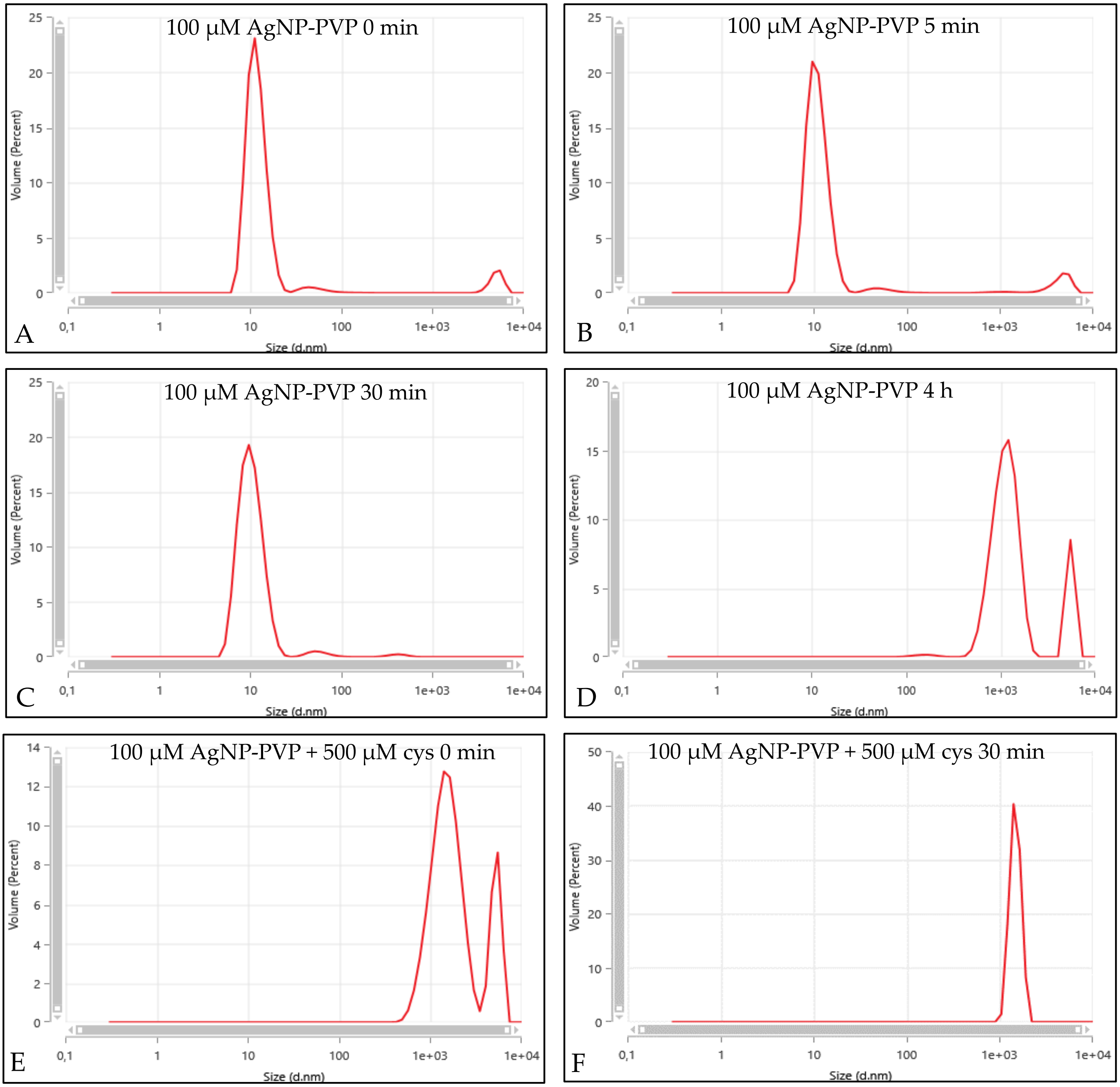

Figure S3. Temporal changes in hydrodynamic diameter of 100 µM AgNP-PVP alone after the addition into liquid ½ strength MS medium; in the beggining of the measurement (A), after five minutes (B), after 30 minutes (C), and after four hours (D); after the addition of 500 µM of cysteine (cys), in the beggining of the measurement (E), and after 30 minutes (F).
