# Supplementary material for: Effects of Silver Nanoparticles on Physiological and Proteomic Responses of Tobacco (Nicotiana tabacum) Seedlings Are Coating-Dependent
Source: Int J Mol Sci. 2022 Dec 14;23(24):15923. doi: 10.3390/ijms232415923 (PMC9787911; doi:10.3390/ijms232415923)
Supplement: Supplementary file 1 [file ijms-23-15923-s001.zip › Figure S5_2DE gels.pdf]

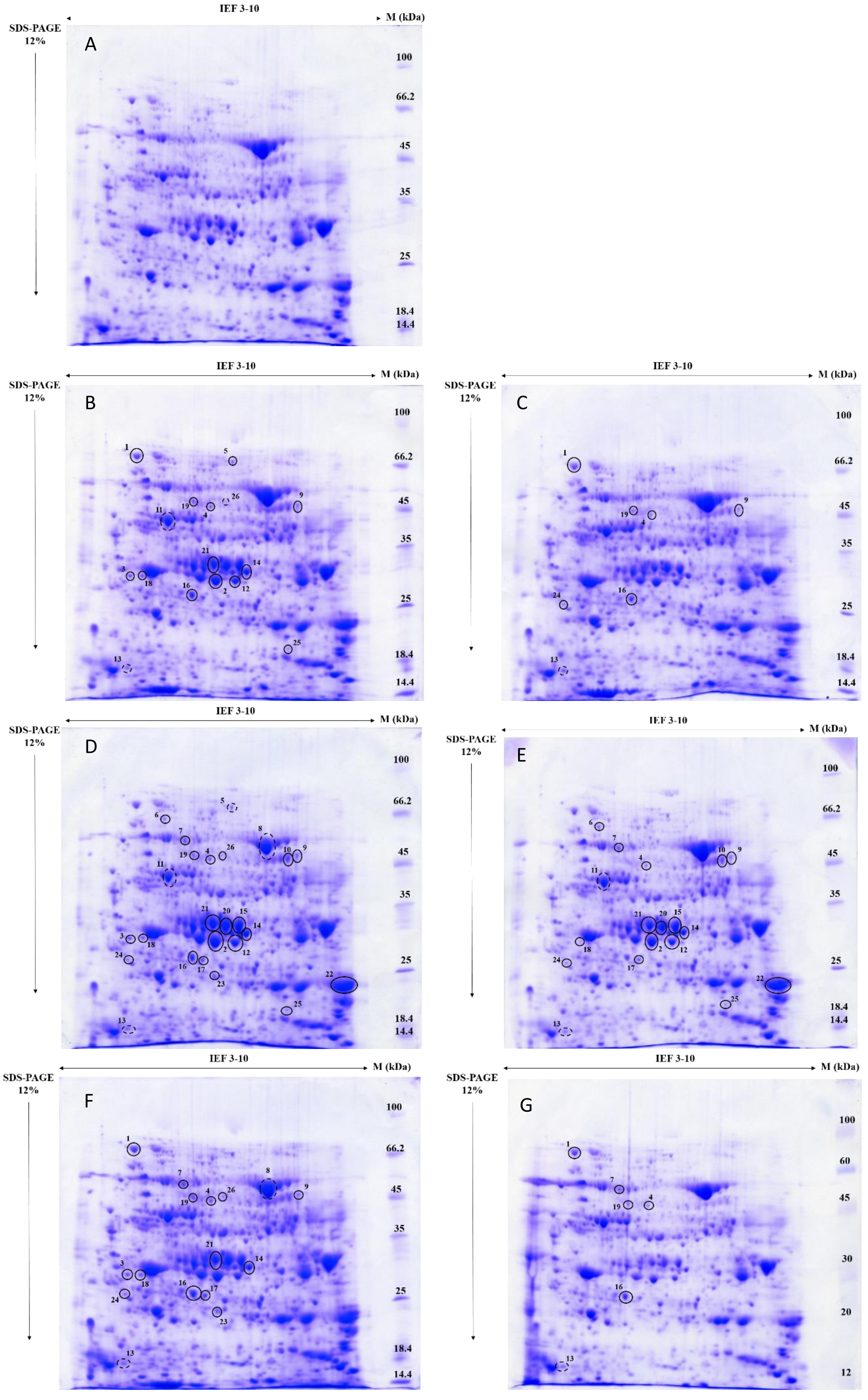

Figure S5. Proteome profiles of control seedlings (A) and seedlings treated with 100 μM AgNP-PVP (B), 100 μM AgNP-PVP and 500 μM cysteine (C), 100 μM AgNP-CTAB (D), 100 μM AgNP-CTAB and 500 μM cysteine (E), 100 μM $AgNO_3$ (F) and 100 μM $AgNO_3$ and 500 μM cysteine (G) obtained after 2-DE analysis. Differently abundant proteins (at least 1.5-fold compared to the control) are indicated by circles; upregulated proteins are indicated by black line, while down-regulated proteins are indicated with broken-lined circles. The numbers correspond to the numbers listed in Table 2. M, molecular weight markers
