# Supplementary material for: Effects of Silver Nanoparticles on Physiological and Proteomic Responses of Tobacco (Nicotiana tabacum) Seedlings Are Coating-Dependent
Source: Int J Mol Sci. 2022 Dec 14;23(24):15923. doi: 10.3390/ijms232415923 (PMC9787911; doi:10.3390/ijms232415923)
Supplement: Supplementary file 1 [file ijms-23-15923-s001.zip › Table S1_zeta potential analysis_revised.pdf]

Table S1. Temporal changes in ζ potential of 100 μM AgNP-PVP and AgNP-CTAB in liquid ½ strength MS medium. Values are means ± SD of five measurements.

	**AgNP-PVP**	**AgNP-CTAB**
**0 min**	-11.2 ± 0.8	-7.0 ± 2.7
**5 min**	-8.7 ± 1.4	-4.9 ± 0.5
**30 min**	-12.7 ± 0.2	-5.3 ± 2.4
**4 h**	-12.1 ± 0.7	-5.2 ± 0.5
